# Supplementary material for: Candidate Genes, Markers, Signatures of Selection, and Quantitative Trait Loci (QTLs) and Their Association with Economic Traits in Livestock: Genomic Insights and Selection
Source: Int J Mol Sci. 2025 Aug 8;26(16):7688. doi: 10.3390/ijms26167688 (PMC12386557; doi:10.3390/ijms26167688)
Supplement: Supplementary file 1 [file ijms-26-07688-s001.zip › ijms-3738479-supplementary.pdf]

Supplementary File S1—Supplementary Tables

Table S1. Identification of QTLs for economically important traits in various livestock species/breeds.

Trait Category	Specific Trait	Phenotype Description	Chromosome(s)	Species/Breed	Key Genes/Markers	Effect Size/Heritability	Strength of Evidence	Breeding Application	Ref.
Carcass Characteristics	Fat Deposition	Back-fat thickness, subcutaneous fat depth	4, 5, 9, 16	Texel sheep	*PPARG, FTO*	Moderate (h^2 = 0.25–0.35)	Grade B	Selecting leaner carcasses	[1]
	Muscling	Muscle depth, rib-eye area	6, 9, 11	Texel sheep, Cattle	*MYF5, MSTN*	High (h^2 = 0.40~0.50)	Grade A	Enhancing meat yield	[1]
	Carcass Weight	Hot carcass weight	6, 9, 10, 18, 29	Texel sheep, Cattle	*IGF2, GHR*	High (h^2 = 0.45)	Grade C	Improving growth efficiency	[2] [3]
Carcass Composition	Meat Quality	Marbling score (intramuscular fat), tenderness	3, 10, 14, 20, 27	Cattle	*DGAT1, CAPN1*	Moderate (h^2 = 0.20–0.30)	Grade A	Premium meat markets	[3]
	Fat Distribution	Kidney, heart, and pelvic fat	15	Cattle	*LEPR, SCD1*	Low (h^2 = 0.15)	Grade C	Reducing waste fat	[3]
Growth & Development	Early Growth	Birth weight, weaning weight	1–4, 8, 16–19, 22, 27–29	Sheep, Cattle, Angora goat	*GH1, GDF8*	High (h^2 = 0.35~0.55)	Grade B	Reducing neonatal mortality	[3] [4] [5]
	Post-Weaning Efficiency	Average daily gain, post-weaning growth	2, 3, 5, 8, 9, 17, 20	Zel sheep, Lori-Bakhtiari sheep, Rayini goat	*IGF1, POU1F1*	Moderate (h^2 = 0.25~0.40)	Grade B	Feed efficiency optimization	[6] [7] [8]
Disease Resistance	Parasite Resistance	Gastrointestinal nematode resistance	1, 3, 5, 7–9, 14, 16, 21–23, 26	Creole goat, Angora goat	*IL2, TLR4*	High (h^2 = 0.30~0.45)	Grade B	Reducing anthelmintic use	[8]
	Mastitis Resistance	Udder health, somatic cell count	6	Cattle	BoLA-DRB3	Moderate (h^2 = 0.25)	Grade B	Improving dairy herd longevity	[9]

Supplementary File S1—Supplementary Tables

**Milk Production**	Yield & Composition	Milk yield, fat percentage, protein percentage	3, 6, 14, 20	Goat, Dairy cattle	*CSN2*, *CSN3*, *DGAT1*	High (h^2 = 0.40~0.60)	Grade A	Tailoring milk for cheese/butter production	[10] [11]
**Fibre Production**	Fibre Traits	Fibre diameter, length, yield	1–5, 8, 13, 18, 20, 24	Angora goat, Rayini goat	*KRTAP*, *FGF5*	Moderate (h^2 = 0.20~0.35)	Grade C	Enhancing luxury fibre markets	[12] [13]

Note: The strength of evidence for each gene or marker association is graded based on the scientific literature. **Grade A:** For functionally validated genes, where the gene's function has been confirmed through molecular or physiological experiments. **Grade B:** For strong associations identified through large-scale Genome-Wide Association Studies (GWAS) with robust statistical support. **Grade C:** For preliminary associations identified in smaller-scale studies, candidate gene approaches, or initial exploratory research.

Supplementary File S1—Supplementary Tables

Table S2. Candidate Genes Associated with Growth, Muscle Development, Body size, and Meat production and quality.

No.	Gene Name	Gene Symbol	Biological Function	Associated Traits	Strength of Evidence	Ref.
1	Growth Hormone	*GH*	Produces growth hormone, essential for regulating birth and weaning weights.	Growth, metabolism, postnatal development, milk yield	Grade B	**[14]** [15] [16]
2	Arylalkylamine N-Acetyltransferase	*AANAT*	Involved in melatonin synthesis, reducing lipid peroxidation and protecting fatty acids.	Fat content, omega-3 PUFAs, flavor characteristics	Grade B	[17-19]
3	Insulin-Like Growth Factor 1	*IGF1*	Stimulates growth and development; influences metabolic pathways in mammals.	Growth rate, reproduction efficiency	Grade A	[20] [21,22]
4	Calpain-1	*CAPN1*	Facilitates protein breakdown in post-mortem muscle; involved in fat metabolism.	Meat tenderness and quality	Grade A	[23-25] [23,26]
5	Leptin	*LEP*	Regulates energy balance and body weight; impacts feed conversion efficiency.	Growth development, metabolic balance	Grade B	[27,28]
6	Calpastatin	*CAST*	Inhibits calpains, affecting muscle structure and fat deposition.	Tenderness, juiciness, colour of meat	Grade A	[17,29] [30,31] [32]
7	Pituitary-Specific Transcription Factor 1	*POU1F1*	Regulates secretion of pituitary hormones; influences muscle and milk production.	Carcass traits, growth performance	Grade A	[33] [34] [33]
8	Caveolin-3	*CAV3*	Key protein in muscle cell membranes; essential for signaling processes.	Muscle fibre development	Grade C	[35] [36] [37]

Supplementary File S1—Supplementary Tables

9	Cell Cycle-Gated Protein 1	*CCNB1*	Regulates cell cycle progression, impacting tissue growth and development.	Carcass yield, muscle growth	Grade C	[38] [39] [40]
10	Corticotrophin-Releasing Hormone	*CRH*	Helps regulate stress responses and metabolic processes.	Carcass weight, overall growth	Grade B	[41] [42]
11	Myostatin	*MSTN*	Inhibits muscle development; mutations can lead to increased muscle mass.	Muscle growth and development candidates	Grade A	[43] [44] [45] [46]
12	Kappa Casein	*CSN3*	Important for milk protein composition and effects on cheese production.	Milk yield, fat and protein content	Grade A	[47] [48]
13	Heat Shock Protein 27 kDa	*HSPB1*	Involved in muscle stress response and protein folding.	Tenderness and quality of meat	Grade C	[49] [50]
14	Pro-opiomelanocortin	*POMC*	Regulates appetite and energy expenditure; influences growth.	Growth rate, carcass yield	Grade C	[42] [51]
15	Bone Morphogenetic Protein	*BMP*	Plays a role in bone formation and tissue repair.	Musculoskeletal development, fertility	Grade B	[52] [53] [54]
16	RAR-related Orphan Receptor Alpha	*RORA*	Involved in lipid metabolism and muscle growth regulation.	Body composition, obesity predisposition	Grade C	[55] [56]
17	Thyroglobulin	*TG*	Precursor to thyroid hormones; crucial for metabolism.	Fat deposition, marbling in meat	Grade C	[57] [58] [59]
18	Fatty Acid Synthase	*FASN*	Facilitates fatty acid synthesis, playing a role in energy metabolism.	Fat content, marbling	Grade A	

Supplementary File S1—Supplementary Tables

19	Carboxypeptidase E	*CPE*	Involved in protein processing and hormone activation.	Muscle quality, flavor profile	Grade C
20	Platelet-Derived Growth Factor	*PDGF*	Promotes cell growth and division; involved in muscle regeneration.	Muscle growth, recovery from injury	
21	Sarcoplasmic Reticulum Ca2+ ATPase	*ATP2A1*	Regulates calcium levels in muscle, essential for muscle contraction and relaxation.	Muscle performance, meat tenderness	Grade C
22	Peroxisome Proliferator-Activated Receptor Gamma	*PPARγ*	Regulates fat storage and glucose metabolism.	Body composition, lipid metabolism	Grade A
23	Estrogen Receptor Alpha	*ESR1*	Mediates effects of estrogen on growth and development.	Muscle development, growth rate	Grade C
24	CD68 Molecule	*CD68*	Marker for macrophages involved in immune response in tissues.	Meat quality, tenderness	Grade C
25	Interleukin 6	*IL6*	Involved in inflammatory responses and muscle metabolism.	Muscle growth, recovery	Grade C
26	Forkhead Box O1	*FOXO1*	Impacts muscle atrophy and metabolism under stress conditions.	Muscle mass maintenance, overall health	Grade A
27	Thyroid-Stimulating Hormone Beta	*TSHB*	Linked to muscular functionality.	Meaty traits, feed conversion	Grade C
28	Neuroblastoma RAS Viral Oncogene Homolog	*NRAS*	Involved in growth and cellular functions.	Meaty traits, feed conversion	Grade C
29	Adenosine Monophosphate Deaminase 1	*AMPD1*	Important in energy metabolism in skeletal muscle.	Meaty traits, feed conversion	Grade C
30	EGF Containing Fibulin-like Extracellular Matrix Protein 1	*EFEMP1*	Linked to conjugated linoleic acid content in meat.	Meat quality	Grade C

31	Lipid and Carbohydrate Metabolism Regulator	*LCORL*	Associated with body size linked to stature in cattle.	Body size	Grade B
32	Tumor Necrosis Factor	*TNF*	Involved in systemic inflammation and plays a role in metabolism.	Body size, overall health	Grade B
33	Tumor Protein P53	*TP53*	Involved in cell cycle regulation and apoptosis.	Body size, overall health	Grade A
34	TGS1, LYN, PLAG1	Various	Linked to stature, reflected in both humans and cattle.	Body size	Grade B
35	A-CHCHD7, SMAD2	Various	Influences body size in humans, cattle, horses, and dogs.	Body size	Grade C

Note: The strength of evidence for each gene or marker association is graded based on the scientific literature. **Grade A:** For functionally validated genes, where the gene's function has been confirmed through molecular or physiological experiments. **Grade B:** For strong associations identified through large-scale Genome-Wide Association Studies (GWAS) with robust statistical support. **Grade C:** For preliminary associations identified in smaller-scale studies, candidate gene approaches, or initial exploratory research.

Supplementary File S1—Supplementary Tables

Table S3. Candidate Genes Implicated in Reproductive Biology: Functions and Associated Traits.

No.	Gene Name	Gene Symbol	Biological Function	Associated Traits	Strength of Evidence	Ref.
**1**	Poly(A) Binding Protein 2B	*PAIP2B*	Neurohypophyseal hormone activity	Reproductive-related processes		
**2**	Coiled-Coil Domain Containing 64	*CCDC64*	Involved in microtubule organization	Reproductive processes	Grade C	[60]
**3**	Erythrocyte Membrane Protein Band 4.1 Like 5	*EPB41L5*	Cell adhesion and signalling	Reproductive health		
**4**	Baculoviral IAP Repeat Containing 6	*BIRC6*	Apoptotic regulation	Development and maintenance of gametes		
**5**	TAO Kinase 1	*TAOK1*	Regulation of cell growth and apoptosis	Reproductive traits	Grade B	[61]
**6**	Chromosome 6 Open Reading Frame 4	*C6H4*	Not well characterized	Potential role in reproductive functions		
**7**	Solute Carrier Family 33 Member 1	*SLC33A1*	Involved in neurotransmitter transport	May influence reproductive signalling		
**8**	Poly(A) Binding Protein 2	*PAIP2*	Implicated in sex determination	Mating type determination	Grade A	[60]
**9**	C-Type Lectin Domain 16A	*CLEC16A*	Immunity and reproduction	Mating preferences		
**10**	Partitioning Defective 3 Homolog B	*PARD3B*	Regulation of cell polarity	Spermatid maturation		
**11**	FAT Tumor Suppressor 1	*FAT1*	Cellular signalling and adhesion	Spermatogenesis	Grade A	[62]
**12**	Lysine Demethylase 4C	*KDM4C*	Histone demethylation	Spermatogenesis		
**13**	Tubulin Polymerization Promoting Protein 3	*TPPP3*	Involved in tubulin stabilization	Male gamete production	Grade C	[63]

Supplementary File S1—Supplementary Tables

**14**	KH Domain-Containing, RNA-Binding, Signal Transduction Associated Protein 2	*KHDRBS2*	Regulates the number of teats and pregnancy status	Numeric trait determination	Grade C	[60]
**15**	Forkhead Box L2	*FOXL2*	Transcription factor for female reproductive activity	Essential for tissue development	Grade A	[64] [65] [66]
**16**	Melatonin Receptor 1A	*MTNR1A*	Melatonin receptor involved in fertility	Seasonal reproduction in mammals	Grade A	[67] [68] [69]
**17**	Sex Determining Region Y	*SRY*	Key gene for sex determination and gonadal development	Testis formation		[69] [70]
**18**	Amelogenin	*AMEL*	Protein vital for tooth development and sex determination	Related to sexual differentiation	Grade A	[71]
**19**	Bone Morphogenetic Protein Receptor 1B	*BMPR1B*	Role in reproductive capacity and litter size	Goat prolificacy	Grade B	
**20**	Growth Differentiation Factor 9	*GDF9*	Oocyte development and function	Female fertility		[72]
**21**	Ectodysplasin A2 Receptor	*EDA2R*	Embryo development and dysplasia	Associated with reproductive anomalies	Grade C	[27]
**22**	High Mobility Group AT-Hook 2	*HMGA2*	Essential for fetal growth	Affects obesity and cryptorchidism	Grade B	[73]
**23**	Membrane-Associated Guanylate Kinase 1	*MAGI1*	Involvement in egg production	Goose reproductive performance	Grade C	[74]
**24**	Nuclear Receptor Subfamily 6 Group A Member 1	*NR6A1*	Regulates oocyte and embryonic development	Critical for reproductive success		[75]
**25**	Androgen Receptor	*AR*	Androgen receptor influencing reproductive organs	Vitality of sperm stores	Grade A	[76]
**26**	Protein Phosphatase 3 Catalytic Subunit A	*PPP3CA*	Regulatory role in meiosis	Associated with reproduction pathways	Grade C	[77]
**27**	Phospholipase C Beta 1	*PLCB1*	Signalling involved in reproduction	GnRH signalling pathway	Grade C	[78,79]

**28**	Serine/Threonine Kinase 3	*STK3*	Signalling involved in oocyte maturation	Related to fertility	Grade C	[80,81]
**29**	Insulin-Like Growth Factor 2 MRNA-Binding Protein 2	*IGF2BP2*	Regulates growth factors in reproductive tissues	Associated with fetal development	Grade C	[82]
**30**	Neuronal Pentraxin 1	*NPTX1*	Neuronal and reproductive signalling	Impacts fertility processes	Grade C	[83]
**31**	Ankyrin Repeat Domain 17	*ANKRD17*	Involved in cellular signalling pathways	Reproductive implications	Grade C	[84]
**32**	C-Cbl-Binding Protein	*CLRB*	Related to immune response and reproduction	Immunological aspects of fertility	Grade C	[85]
**33**	Dihydropyrimidine Dehydrogenase	*DPYD*	Involved in metabolic pathways related to reproduction	Metabolic adaptation during reproduction	Grade C	[86]

Note: The strength of evidence for each gene or marker association is graded based on the scientific literature. **Grade A:** For functionally validated genes, where the gene's function has been confirmed through molecular or physiological experiments. **Grade B:** For strong associations identified through large-scale Genome-Wide Association Studies (GWAS) with robust statistical support. **Grade C:** For preliminary associations identified in smaller-scale studies, candidate gene approaches, or initial exploratory research.

Supplementary File S1—Supplementary Tables

Table S4. Candidate Genes Associated with Milk Production and Composition in Livestock.

Gene Name	Gene Symbol	Chromosome (CHR)	Biological Function	Associated Traits	Species/Breeds	Strength of Evidence	Ref.
EGF Containing Fibulin-Like Extracellular Matrix Protein 1	*EFEMP1*	4 regions including CHR-11, 13, and 14	Involved in the formation of extracellular matrix	Implicated in milk production	-	Grade C	[87] [88]
Pituitary-Specific Positive Transcription Factor	*POU1F1*	1q21-q22	Regulates growth hormone expression	Influences lactation	-	Grade A	[89]
Leptin	*LEP*	3q33	Regulates energy balance and body weight	Associated with feed efficiency	-	Grade B	[90]
Casein Gene Cluster	-	6 between 85.95 and 86.25 Mb	Major component of milk protein	Milk composition and protein yield	Cattle, Sheep, and Goats	Grade A	[91] [92]
Alpha S1 Casein	*CSN1S1*	6 between 75-120 Mb	Component of casein proteins, aids in nutrient absorption	Efficiency of protein synthesis	Dairy Cattle, Goat breeds	Grade A	[93]
Casein Alpha S2	*CSN1S2*	-	Component of casein proteins	High protein percentage and cheese yield	-	Grade A	[94]
Casein Beta 2	*CSN2*	-	Affects calcium sensitivity and protein levels	Sensitivity to calcium	-	Grade A	[95]
Casein Alpha 3	*CSN3*	-	Contributes to casein content	Associated with casein content	-	Grade A	[93]
ATP Binding Cassette Sub-Family G Member 2	*ABCG2*	6	Involved in transport of lipids and drugs	Milk composition and yield	Murciano, Granadina, Argentata, dell'Etna	Grade B	[96]
Candidate regions	-	1 between 110 and 130 Mb	Associated with dairy productivity	Dairy and Brahman cattle	-		-
Lysophospholipase I	*LYPLA1*	-	Involved in lipid metabolism	Feed intake and weight gain	-	Grade B	[97]
Regulator of G-Protein Signaling 20	*RGS20*	-	Involved in hormonal regulation	Influences growth and development	-	Grade C	[98]

Supplementary File S1—Supplementary Tables

SRY-Box Transcription Factor 17	*SOX17*	-	Important for cell differentiation	Impacts reproductive development	-		-
Fatty Acid Transporter 1	*SLC27A1*	7; 0.037 Mb	Mediates fatty acid uptake in tissues	Fat content in milk	Saanen and Alpine breeds	Grade B	[99]
Diacylglycerol O-acyltransferase 1	*DGAT1*	14; 0.111 Mb	Catalyzes the final step of triacylglycerol synthesis	-	-		-
Bovine Alpha Lactalbumin Precursor	*PAEP*	11; 0.034 Mb	Influences lactose synthesis in mammary gland	Protein content in milk	-		-
Phospholipase D2	*PLD2*	19; 0.258 Mb	Involved in lipid metabolism	Protein yield in milk	-		-
Arachidonate 12-Lipoxygenase	*ALOX12*	19; 0.258 Mb	Involved in eicosanoid synthesis	Protein yield in milk	-		-
Gamma-Glutamyl Transferase 6	*GGT6*	19; 0.258 Mb	Part of glutathione metabolism	Protein yield in milk	-		-
Arachidonate 15-Lipoxygenase	*ALOX15*	19; 0.258 Mb	Regulates inflammation and lipid metabolism	Protein yield in milk	-		-
Arachidonate 12B-Lipoxygenase	*ALOX12B*	19; 0.258 Mb	Involved in fatty acid metabolism	Protein yield in milk	-		-
Candidate regions across multiple chromosomes	-	11 regions on 8 chromosomes	Various genetic contributions to milk production	Milk production	Several dairy goat breeds	Grade B	
Candidate regions and/or SNPs	-	286 regions across all autosomes	Genetic variation associated with milk traits	-	-		[87] [88]
Candidate regions over multiple chromosomes	-	24 regions on 15 chromosomes	-	-	-		

Note: The strength of evidence for each gene or marker association is graded based on the scientific literature. Grade A: For functionally validated genes, where the gene's function has been confirmed through molecular or physiological experiments. Grade B: For strong associations identified through large-scale Genome-Wide Association Studies (GWAS) with robust statistical support. Grade C: For preliminary associations identified in smaller-scale studies, candidate gene approaches, or initial exploratory research.

Supplementary File S1—Supplementary Tables

Table S5. Candidate Genes and SS Associated with Fibre Production, Coat Color and Skin Sensitivity.

No.	Gene Name	Gene Symbol	Chromosome (CHR)	Biological Function	Associated Traits	Species/Breeds	Strength of Evidence	Ref.
1	Cut-like 1	*CUX1*	25 (34.69-36.43 Mb)	Involved in hair texture and curl pattern	Wavy hair, curly whiskers	Angora, Ankara	Grade A	[100]
2	Procollagen-Lysine, 2-Oxoglutarate 5-Dioxygenase 3	*PLOD3*	25 (34.69-36.43 Mb)	Essential for collagen formation	Hair texture	Angora, Ankara	Grade A	[101]
3	Disintegrin and Metalloproteinase with Thrombospondin Motifs 20	*ADAMTS20*	5 (70.0–70.5 Mb)	Important for melanoblast survival	Coat colour, skin sensitivity	Angora, Ankara, Kil, Kilis	Grade B	[102]
4	Metalloproteinase Inhibitor 3	*TIMP3*	5 (70.0–70.5 Mb)	Regulates matrix metalloproteinases	Skin health	Angora, Ankara, Kil, Kilis	Grade C	[102]
5	SRY-Box Transcription Factor 18	*SOX18*	13 (53.0–53.5 Mb)	Critical for hair follicle development	Hair follicle health	Various breeds	Grade A	[103]
6	Melanocortin 1 Receptor	*MC1R*	18 (15.50–16.25 Mb)	Regulates pigmentation (eumelanin/pheomelan in switch)	Coat colour	Various breeds	Grade A	[104]
7	Dual Specificity Protein Phosphatase 22	*DUSP22*	23 (0.025–0.35 Mb)	Affects pigmentation and skin sensitivity	Coat colour	Humans, mice, Meigu goat		
8	Interferon Regulatory Factor 4	*IRF4*	SS	Regulates melanin production	Coat colour	Humans, mice	Grade A	-105] [108
9	Exocyst Complex Component 2	*EXOC2*	SS	Influences exocytosis related to pigmentation	Skin sensitivity	Various breeds	Grade C	
10	Keratin 81	*KRT81*	17 (1.5–1.6 Mb)	Plays a role in hair fibre structure	Fibre production	Various breeds		

Supplementary File S1—Supplementary Tables

11	Fibroblast Growth Factor 5	*FGF5*	21 (3.5–4.0 Mb)	Influences hair follicle development	Hair length	Various breeds		
12	Oligophrenin 1	*OFC1*	19 (30.0–30.5 Mb)	Associated with hair texture	Hair structure	Angora goats, humans		
13	Solute Carrier Family 45 Member 2	*SLC45A2*	15 (7.6–7.8 Mb)	Involved in pigmentation regulation	Coat colour	Various breeds		
14	Tyrosinase	*TYR*	11 (43.5–43.7 Mb)	Involved in melanin synthesis	Coat colour	Various species		
15	Tyrosinase-Related Protein 1	*TYRP1*	9 (50.5–51.0 Mb)	Associated with melanin production	Coat colour	Various breeds		
16	Family with Sequence Domain 83 Member G	*FAM83G*	20 (0.02–0.03 Mb)	Potential role in hair growth	Hair follicle health	Various breeds		
17	Fibroblast Growth Factor 7	*FGF7*	12 (104.5–105.0 Mb)	Involved in hair follicle development	Hair growth	Various breeds	Grade C	[109,110]
18	Unknown Region 1	SS	5 (36.25–36.75 Mb)	Linked to coat colour and sensitivity	Coat colour, skin sensitivity	Various breeds		
19	Unknown Region 2	SS	9 (11.5–12 Mb)	-	-	-		
20	Unknown Region 3	SS	13 (53.0–53.5 Mb, 62.75–63.25 Mb)	-	-	-	Grade B	
21	KIT	SS	-	Linked to white coat phenotype	White-coat	Holstein cattle		[109,110]
22	Unknown Region 5	SS	8 (27.0–27.5 Mb)	-	-	Angora, Ankara		
23	Unknown Region 6	SS	22 (2.25–3.0 Mb)	-	-	-		
24	Unknown Region 7	SS	29 (39.25–39.75 Mb)	-	-	-		

Note: The strength of evidence for each gene or marker association is graded based on the scientific literature. **Grade A:** For functionally validated genes, where the gene's function has been confirmed through molecular or physiological experiments. **Grade B:** For strong associations identified through large-scale Genome-Wide Association Studies (GWAS) with robust statistical support. **Grade C:** For preliminary associations identified in smaller-scale studies, candidate gene approaches, or initial exploratory research.

Supplementary File S1—Supplementary Tables

Table S6. Candidate Genes Associated with Adaptation Traits, Disease Resistance, Heat Tolerance and Stress Response in Livestock.

No.	Gene Name	Gene Symbol	Biological Function (Inferred)	Associated Traits	Breed	Strength of Evidence	Ref.
1	Superoxide dismutase 1	*SOD1*	Antioxidant defense; protects cells from oxidative stress.	Thermoregulation	Bos indicus	Grade A	[111]
2	DnaJ heat shock protein family member C8	*DNAJC8*	Molecular chaperone; assists in protein folding under stress.	Heat-Tolerance	East African Shorthorn Zebu	Grade A	[112]
3	DnaJ heat shock protein family member C8	*DNAJC8*	Molecular chaperone; assists in protein folding under stress.	Heat-Tolerance	East African Shorthorn Zebu	Grade A	[113]
4	Oxytocin receptor	*OLAI*	Oxytocin Receptor	Heat-Tolerance	Muturu	Grade C	[114]
5	NADH: ubiquinone oxidoreductase subunit B3 and DIS3 like exonuclease 2	*NDUFB3, DIS3L2*	NDUFB3: Part of mitochondrial respiratory chain; DIS3L2: RNA exosome complex component.	Adaptation to environmental conditions	Holstein-Friesian	Grade C	[115]
6	Serpin family E member 2	*SERPINE2*	Serine protease inhibitor; regulates blood coagulation and tissue remodelling.	Heat Stress Response	Holstein-Friesian	Grade C	[116]
7	Sperm-associated antigen 17	*SPAG17*	Involved in sperm motility; important for flagellar function.	Heat Stress Response	Holstein-Friesian	Grade C	[117]
8	Sperm-associated antigen 17	*SPAG17*	Involved in sperm motility; important for flagellar function.	Rectal Temperature	Holstein-Friesian	Grade C	[118]
9	Cytidine monophosphate kinase 1	*CMPK1*	Cytidine monophosphate kinase; involved in nucleotide metabolism.	Heat-Tolerance	Zebu	Grade C	[119]
10	Prolactin-releasing hormone	*PRLH*	Prolactin-releasing hormone; stimulates prolactin secretion.	Thermoregulation	Bos indicus	Grade B	[111]
11	Melanophilin and Rab17	*MLPH, RAB17*	MLPH: Involved in melanosome transport and pigmentation; RAB17: Involved in vesicular trafficking.	Thermoregulation	African cattle	Grade A	[120]
12	DnaJ heat shock protein family member B4	*DNAJB4*	Molecular chaperone; assists in protein folding.	Heat-Tolerance	Ongole	Grade A	[121]

Supplementary File S1—Supplementary Tables

13	Hydroxysteroid 17-beta dehydrogenase 7	*HSD17B7*	Hydroxysteroid 17-beta dehydrogenase; involved in steroid hormone metabolism.	Respiration Rate	Holstein-Friesian	Grade B	[116]
14	Kelch repeat and BTB domain containing 2 and LSM5 homolog	*KBTBD2, LSM5*	KBTBD2: Involved in protein-protein interaction; LSM5: Component of the Sm complex involved in RNA splicing.	Rectal Temperature	Holstein-Friesian	Grade B	[122]
15	Metabotropic glutamate receptor 8	*GRM8*	Metabotropic glutamate receptor; involved in synaptic transmission.	Heat-Tolerance	Holstein-Friesian	Grade C	[123]
16	Acid-sensing ion channel 3	*ASIC3*	Acid-sensing ion channel; involved in pain sensation and mechanotransduction.	Heat Stress Response	African cattle	Grade B	[124]
17	Cystic fibrosis transmembrane conductance regulator	*CFTR*	Chloride channel; regulates ion and water transport across epithelial cells.	Thermoregulation	African cattle	Grade A	[120]
18	Aquaporin 1	*AQP1*	Aquaporin; involved in water transport across cell membranes.	Adaptation to environmental conditions	Holstein-Friesian	Grade A	[115]
19	Cyclin-dependent kinase inhibitor 1B	*CDKN1B*	Cyclin-dependent kinase inhibitor; regulates cell cycle progression.	Thermotolerance	Holstein-Friesian	Grade C	[125]
20	Malonyl-CoA acyl carrier protein transacylase	*MCAT*	Malonyl-CoA-acyl carrier protein transacylase; involved in fatty acid synthesis.	Heat-Tolerance	Holstein-Friesian	Grade C	[126]
21	Solute carrier family 1 member C1 and Phosphodiesterase 3A	*SLCO1C1, PDE3A*	SLCO1C1: Transports thyroid hormones; PDE3A: Involved in cAMP signalling and smooth muscle relaxation.	Rectal Temperature	Holstein-Friesian	Grade C	[122]
22	FK506 binding protein 4	*FKBP4*	Peptidyl-prolyl cis-trans isomerase; chaperone protein involved in protein folding.	Heat-Tolerance	Gir	Grade A	[121]
23	DnaJ heat shock protein family member 14	*DNAJC14*	Molecular chaperone; may assist in protein folding.	Heat-Tolerance	East African Shorthorn Zebu	Grade A	[113]

Supplementary File S1—Supplementary Tables

24	Homeobox C12, Homeobox C13 and Inositol trisphosphate receptor 2	*HOXC12, HOXC13, ITPR2*	HOXC12/13: Transcription factors involved in development; ITPR2: Inositol trisphosphate receptor, involved in calcium signaling.	Thermoregulation	African cattle	Grade C	[120]
25	Transmembrane protein 33	*TMEM33*	Transmembrane protein; function poorly characterized.	Heat Stress Response	Holstein-Friesian	Grade C	[117]
26	Neuropeptide FF receptor 2	*NPFFR2*	Neuropeptide FF receptor; involved in pain modulation and stress response.	Heat-Tolerance	Holstein-Friesian	Grade C	[127]
27	Glutaredoxin-related protein 1	*GRXCR1*	Glutaredoxin-related protein; involved in redox homeostasis.	Heat-Tolerance	Tharparkar	Grade A	[121]
28	Heat shock protein family A member 4	*HSPA4*	Molecular chaperone; assists in protein folding and prevents aggregation under stress.	Heat-Tolerance	Zebu	Grade A	[119]
29	Heat shock protein family A member 9	*HSPA9*	Molecular chaperone; assists in protein folding and prevents aggregation under stress.	Heat-Tolerance	Bos indicus	Grade A	[111]
30	DnaJ heat shock protein family member 18	*DNAJC18*	Molecular chaperone; may assist in protein folding.	Heat-Tolerance	East African Shorthorn Zebu	Grade A	[112]
31	DnaJ heat shock protein family member 18	*DNAJC18*	Molecular chaperone; may assist in protein folding.	Heat-Tolerance	East African Shorthorn Zebu	Grade A	[113]
32	Toll-like receptor 4	*TLR4*	Toll-like receptor; involved in immune response and inflammation.	Heat-Tolerance	Holstein-Friesian	Grade A	[123]
33	Interleukin 6	*IL6*	Interleukin-6; cytokine involved in inflammatory response.	Heat-Tolerance	Ongole	Grade A	[121]
34	Regulator of G-protein signaling 3	*RGS3*	Regulator of G-protein signaling; modulates G-protein-coupled receptor signaling.	Adaptation to environmental conditions	Holstein-Friesian	Grade B	[115]
35	Reticulon 4 interacting protein 1	*RTN4IP1*	Reticulon-4 interacting protein; involved in endoplasmic reticulum organization.	Respiration Rate	Holstein-Friesian	Grade C	[117]

Supplementary File S1—Supplementary Tables

36	Serum/glucocorticoid regulated kinase 1	*SGK1*	Serum/glucocorticoid regulated kinase; involved in ion transport and cell survival.	Thermoregulation	African cattle	Grade B	[120]
37	RNA-binding motif protein 25	*RBM25*	RNA-binding motif protein; involved in RNA processing and splicing.	Heat Stress Response	Holstein Friesian	Grade C	[128]
38	SMAD family member 3	*SMAD3*	SMAD family member; intracellular signal transducer in the TGF-beta signaling pathway.	Heat Tolerance	Holstein-Friesian	Grade B	[123]
39	Serine palmitoyltransferase long-chain base subunit	*SPTLC2*	Serine palmitoyltransferase long chain base subunit; involved in sphingolipid biosynthesis.	Adaptation to environmental conditions	Holstein-Friesian	Grade C	[115]
40	Progesterone-associated endometrial protein and Epidermal Pickles protein	*PAEP, EPPK1*	PAEP: Progesterone-associated endometrial protein; EPPK1: Epidermal Pickles protein.	Unsaturated Fatty Acids	Holstein-Friesian	Grade C	[129]
41	Sodium/hydrogen exchanger 4	*SLC9A4*	Sodium/hydrogen exchanger; involved in intracellular pH regulation.	Thermoregulation	African cattle	Grade B	[120]
42	Heat shock protein family H1	*HSPH1*	Molecular chaperone; assists in protein folding and prevents aggregation under stress.	Heat Stress Response	African cattle	Grade A	[124]
43	Integrator complex subunit 6	*INTS6*	Integrator complex subunit; involved in RNA processing.	Heat-Tolerance	Muturu	Grade C	[118]
44	Protein of unknown function	*FAM107B*	Protein of unknown function.	Rectal Temperature	Holstein-Friesian	Grade C	[118]
45	TSNARE1 and RALYL	*TSNARE1, RALYL*	TSNARE1: Involved in membrane fusion; RALYL: RNA processing associated protein.	Rectal Temperature	Holstein-Friesian	Grade C	[118]
46	Heat shock factor 1	*HSF1*	Heat shock factor; transcription factor that regulates heat shock gene expression.	Heat Tolerance	Holstein-Friesian	Grade C	[127]
47	Heat shock factor 1	*HSF1*	Heat shock factor; transcription factor that regulates heat shock gene expression.	Thermotolerance	Holstein-Friesian	Grade B	[125]

48	Heat shock factor 1	*HSF1*	Heat shock factor; transcription factor that regulates heat shock gene expression.	Heat-Tolerance	Holstein-Friesian	Grade A	[126]
49	Heat shock factor 1	*HSF1*	Heat shock factor; transcription factor that regulates heat shock gene expression.	Heat-Tolerance	Zebu	Grade A	[119]
50	Glutamate receptor ionotropic 4	*GRIA4*	Glutamate receptor; involved in excitatory neurotransmission.	Heat-Tolerance	Holstein-Friesian	Grade C	[127]
51	Mitogen-activated protein kinase 8 interacting protein	*MAPK8IP1*	Mitogen-activated protein kinase 8 interacting protein; involved in MAPK signaling.	Thermotolerance	Holstein-Friesian	Grade B	[125]
52	Progesterone receptor	*PGR*	Progesterone receptor; nuclear receptor that mediates the effects of progesterone.	Rectal Temperature	Holstein-Friesian	Grade B	[116]
53	Calcitonin receptor and Growth hormone receptor	*CALCR, GHR*	CALCR: Calcitonin receptor; GHR: Growth hormone receptor.	Heat-Tolerance	Holstein-Friesian	Grade B	[127]
54		*SNORA19, RFWD2, SCARNA3, CEP170, PLD5*	Various functions related to RNA processing, ubiquitination, and protein localization.	Rectal Temperature	Holstein-Friesian	Grade C	[122]
55	Mechanistic target of rapamycin	*MTOR*	Mechanistic target of rapamycin; serine/threonine kinase involved in cell growth and metabolism.	Heat-Tolerance	Zebu	Grade A	[119]
56	Sodium channel epithelial 1 subunit delta	*SCNN1D*	Sodium channel subunit; involved in sodium transport across epithelial cells.	Thermoregulation	African cattle	Grade B	[120]
57	Peroxisomal biogenesis factor 14	*PEX14*	Peroxisomal biogenesis factor; involved in peroxisome assembly.	Adaptation to environmental conditions	Holstein-Friesian	Grade C	[115]
58	Gamma-aminobutyric acid transporter	*GATB*	Gamma-aminobutyric acid transporter; involved in GABA transport.	Respiration Rate	Holstein-Friesian	Grade C	[117]
59	Leukemia inhibitory factor, Oncostatin M,	*LIF, OSM, TXNRD2, DGCR8*	Various functions related to cytokine signaling, redox	ART (Reproductive technologies)	Gir x Holstein Friesian	Grade C	[130]

	Thioredoxin reductase, and DGCR8		regulation, and microRNA biogenesis.				
60	Mevalonate decarboxylase	*MVD*	Mevalonate decarboxylase; involved in cholesterol biosynthesis.	Heat Stress Response	African cattle	Grade C	[124]
61	LUC7-like mRNA processing factor 3	*LUC7L3*	LUC7-like mRNA processing factor; involved in mRNA splicing.	Heat Stress Response	Holstein-Friesian	Grade C	[128]
62	DnaJ heat shock protein family member 7	*DNAJC7*	Molecular chaperone; may assist in protein folding.	Heat-Tolerance	East African Shorthorn Zebu	Grade A	[131]

Note: The strength of evidence for each gene or marker association is graded based on the scientific literature. **Grade A:** For functionally validated genes, where the gene's function has been confirmed through molecular or physiological experiments. **Grade B:** For strong associations identified through large-scale Genome-Wide Association Studies (GWAS) with robust statistical support. **Grade C:** For preliminary associations identified in smaller-scale studies, candidate gene approaches, or initial exploratory research.

Supplementary File S1—Supplementary Tables

References:

1. Hernandez, D.G.; Mucha, S.; Banos, G.; Kaseja, K.; Moore, K.; Lambe, N.; Yates, J.; Bunger, L. Analysis of single nucleotide polymorphisms variation associated with important economic and computed tomography measured traits in Texel sheep. *animal* **2018**, *12*, 915-922.
2. Armstrong, E.; Ciappesoni, G.; Iriarte, W.; Da Silva, C.; Macedo, F.; Navajas, E.; Brito, G.; San Julián, R.; Gimeno, D.; Postiglioni, A. Novel genetic polymorphisms associated with carcass traits in grazing Texel sheep. *Meat science* **2018**, *145*, 202-208.
3. Casas, E.; Shackelford, S.; Keele, J.; Koohmaraie, M.; Smith, T.; Stone, R. Detection of quantitative trait loci for growth and carcass composition in cattle. *Journal of animal science* **2003**, *81*, 2976-2983.
4. Visser, C.; Van Marle-Köster, E.; Snyman, M.; Bovenhuis, H.; Crooijmans, R. Quantitative trait loci associated with pre-weaning growth in South African Angora goats. *Small Ruminant Research* **2013**, *112*, 15-20.
5. Riggio, V.; Matika, O.; Pong-Wong, R.; Stear, M.; Bishop, S. Genome-wide association and regional heritability mapping to identify loci underlying variation in nematode resistance and body weight in Scottish Blackface lambs. *Heredity* **2013**, *110*, 420-429.
6. Zhang, L.; Liu, J.; Zhao, F.; Ren, H.; Xu, L.; Lu, J.; Zhang, S.; Zhang, X.; Wei, C.; Lu, G. Genome-wide association studies for growth and meat production traits in sheep. *PloS one* **2013**, *8*, e66569.
7. Jalil-Sarghale, A.; Shahrbabak, M.M.; Sharbabak, H.M.; Sadeghi, M.; Mura, M. Association of pituitary specific transcription factor-1 (POU1F1) gene polymorphism with growth and biometric traits and blood metabolites in Iranian Zel and Lori-Bakhtiari sheep. *Molecular biology reports* **2014**, *41*, 5787-5792.
8. Abadi, M.M.; Askari, N.; Baghizadeh, A.; Esmailizadeh, A. A directed search around caprine candidate loci provided evidence for microsatellites linkage to growth and cashmere yield in Rayini goats. *Small Ruminant Research* **2009**, *81*, 146-151.
9. Hiendleder, S.; Thomsen, H.; Reinsch, N.; Bennewitz, J.; Leyhe-Horn, B.; Looft, C.; Xu, N.; Medjugorac, I.; Russ, I.; Kühn, C. Mapping of QTL for body conformation and behavior in cattle. *Journal of Heredity* **2003**, *94*, 496-506.
10. Roldán, D.; Rabasa, A.; Saldaño, S.; Holgado, F.; Poli, M.; Cantet, R. QTL detection for milk production traits in goats using a longitudinal model. *Journal of Animal Breeding and Genetics* **2008**, *125*, 187-193.
11. Zhang, Q.; Boichard, D.; Hoeschele, I.; Ernst, C.; Eggen, A.; Murkve, B.; Pfister-Genskow, M.; Witte, L.A.; Grignola, F.E.; Uimari, P. Mapping quantitative trait loci for milk production and health of dairy cattle in a large outbred pedigree. *Genetics* **1998**, *149*, 1959-1973.
12. Cano, E.; Marrube, G.; Roldán, D.L.; Bidinost, F.; Abad, M.; Allain, D.; Vaiman, D.; Taddeo, H.; Poli, M.A. QTL affecting fleece traits in Angora goats. *Small Ruminant Research* **2007**, *71*, 158-164.
13. Visser, C.; Van Marle-Köster, E.; Bovenhuis, H.; Crooijmans, R. QTL for mohair traits in South African Angora goats. *Small ruminant research* **2011**, *100*, 8-14.

14. Li, M.; Min, L.; Sun, G.; Pan, Q.; Shen, W.; Wang, G. Polymorphism analysis of the goat growth hormone gene in the 5'regulatory sequence. *Yi chuan= Hereditas* **2004**, *26*, 831-835.
15. Reinecke, R.; Barnes, M.; Akers, R.; Pearson, R. Effect of selection for milk yield on lactation performance and plasma growth hormone, insulin and IGF-1 in first lactation Holstein cows. *J. Dairy Sci* **1993**, *76*, 286-292.
16. Missohou, A.; Talaki, E.; Laminou, I.M. Diversity and genetic relationships among seven West African goat breeds. *Asian-australasian journal of animal sciences* **2006**, *19*, 1245-1251.
17. Dunner, S.; Sevane, N.; García, D.; Cortés, O.; Valentini, A.; Williams, J.; Mangin, B.; Cañón, J.; Levéziel, H.; Consortium, G. Association of genes involved in carcass and meat quality traits in 15 European bovine breeds. *Livestock Science* **2013**, *154*, 34-44.
18. Fagali, N.; Catalá, A. The effect of melatonin and structural analogues on the lipid peroxidation of triglycerides enriched in ω-3 polyunsaturated fatty acids. *Life sciences* **2007**, *81*, 299-305.
19. Perez, R.; Cañón, J.; Dunner, S. Genes associated with long-chain omega-3 fatty acids in bovine skeletal muscle. *Journal of applied genetics* **2010**, *51*, 479-487.
20. Zhang, C.; Zhang, W.; Luo, H.; Yue, W.; Gao, M.; Jia, Z. A new single nucleotide polymorphism in the IGF-I gene and its association with growth traits in the Nanjiang Huang goat. *Asian-Australasian Journal of Animal Sciences* **2008**, *21*, 1073-1079.
21. Shapiro, L.J.; Shapiro, D.B. Low Anabolic Profile in Assessing a Patient's Overall Hair Loss. In *Practical Aspects of Hair Transplantation in Asians*; Springer: 2018; pp. 687-698.
22. Bale, L.K.; Conover, C.A. Regulation of insulin-like growth factor binding protein-3 messenger ribonucleic acid expression by insulin-like growth factor I. *Endocrinology* **1992**, *131*, 608-614.
23. White, S.; Casas, E.; Wheeler, T.; Shackelford, S.; Koohmaraie, M.; Riley, D.; Chase Jr, C.; Johnson, D.; Keele, J.; Smith, T. A new single nucleotide polymorphism in CAPN1 extends the current tenderness marker test to include cattle of Bos indicus, Bos taurus, and crossbred descent. *Journal of animal science* **2005**, *83*, 2001-2008.
24. Patel, Y.M.; Lane, M.D. Role of calpain in adipocyte differentiation. *Proceedings of the National Academy of Sciences* **1999**, *96*, 1279-1284.
25. Orho-Melander, M.; Klannemark, M.; Svensson, M.K.; Ridderstråle, M.; Lindgren, C.M.; Groop, L. Variants in the calpain-10 gene predispose to insulin resistance and elevated free fatty acid levels. *Diabetes* **2002**, *51*, 2658-2664.
26. Page, B.; Casas, E.; Heaton, M.; Cullen, N.; Hyndman, D.; Morris, C.; Crawford, A.; Wheeler, T.; Koohmaraie, M.; Keele, J. Evaluation of single-nucleotide polymorphisms in CAPN1 for association with meat tenderness in cattle. *Journal of animal science* **2002**, *80*, 3077-3085.
27. Housekencht, C.; Baile, R.; Matteri, L.; Spurlock, M. The biology of leptin: a review. *J. Anim. Sci* **1998**, *76*, 1405-1425.
28. Whitley, N.C.; Walker, E.; Harley, S.; Keisler, D.; Jackson, D. Correlation between blood and milk serum leptin in goats and growth of their offspring. *Journal of animal science* **2005**, *83*, 1854-1859.
29. Barendse, W. DNA markers for meat tenderness. Patent PCT filed 8 February 2002. *US Patent Application 20040115678* **2002**.

30. Casas, E.; White, S.; Wheeler, T.; Shackelford, S.; Koohmaraie, M.; Riley, D.; Chase Jr, C.; Johnson, D.; Smith, T. Effects of calpastatin and μ-calpain markers in beef cattle on tenderness traits. *Journal of Animal Science* **2006**, *84*, 520-525.
31. Reardon, W.; Mullen, A.; Sweeney, T.; Hamill, R. Association of polymorphisms in candidate genes with colour, water-holding capacity, and composition traits in bovine M. longissimus and M. semimembranosus. *Meat science* **2010**, *86*, 270-275.
32. Riaz, M.N.; Ghaffar, A.; Khan, M.F.U. Calpastatin (CAST) gene polymorphism and its association with average daily weight gain in Balkhi and Kajli sheep and Beetal goat breeds. *Pakistan Journal of Zoology* **2012**, *44*.
33. Lan, X.; Pan, C.; Chen, H.; Zhang, C.; Li, J.; Zhao, M.; Lei, C.; Zhang, A.; Zhang, L. An AluI PCR-RFLP detecting a silent allele at the goat POU1F1 locus and its association with production traits. *Small Ruminant Research* **2007**, *73*, 8-12.
34. Li, S.; Crenshaw, E.B.; Rawson, E.J.; Simmons, D.M.; Swanson, L.W.; Rosenfeld, M.G. Dwarf locus mutants lacking three pituitary cell types result from mutations in the POU-domain gene pit-1. *Nature* **1990**, *347*, 528.
35. Parton, R.; Way, M.; Stang, E. Caveolin-3 associates with developing T-tubules during muscle differentiation: Evidence for a role for caveolins in plasma membrane morphogenesis. *Molecular Biology of the Cell* **1996**, *7*, 1963-1963.
36. Le Lay, S.; Kurzchalia, T.V. Getting rid of caveolins: phenotypes of caveolin-deficient animals. *Biochimica et Biophysica Acta (BBA)-Molecular Cell Research* **2005**, *1746*, 322-333.
37. Williams, T.M.; Lisanti, M.P. The Caveolin genes: from cell biology to medicine. *Annals of medicine* **2004**, *36*, 584-595.
38. Singh, U.; Roswall, P.; Uhrbom, L.; Westermark, B. CGGBP1 regulates cell cycle in cancer cells. *BMC molecular biology* **2011**, *12*, 28.
39. Singh, U.; Westermark, B. CGGBP1 is a nuclear and midbody protein regulating abscission. *Experimental cell research* **2011**, *317*, 143-150.
40. Patel, D.; Patel, M.; Westermark, B.; Singh, U. Dynamic bimodal changes in CpG and non-CpG methylation genome-wide upon CGGBP1 loss-of-function. *BMC research notes* **2018**, *11*, 419.
41. Solinas, G.; Summermatter, S.; Mainieri, D.; Gubler, M.; Montani, J.-P.; Seydoux, J.; Smith, S.; Dulloo, A.G. Corticotropin-releasing hormone directly stimulates thermogenesis in skeletal muscle possibly through substrate cycling between de novo lipogenesis and lipid oxidation. *Endocrinology* **2006**, *147*, 31-38.
42. Buchanan, F.; Thue, T.; Yu, P.; Winkelman-Sim, D. Single nucleotide polymorphisms in the corticotrophin-releasing hormone and pro-opiomelancortin genes are associated with growth and carcass yield in beef cattle. *Animal genetics* **2005**, *36*, 127-131.
43. Li, X.; Wu, Z.L.; Gong, Y.; Liu, Y.; Liu, Z.; Wang, X.; Xin, T.; Ji, Q. Single-nucleotide polymorphism identification in the caprine myostatin gene. *Journal of Animal Breeding and Genetics* **2006**, *123*, 141-144.
44. Miranda, M.; Amigues, Y.; Boscher, M.; Ménissier, F.; Cortés, O.; Dunner, S. Simultaneous genotyping to detect myostatin gene polymorphism in beef cattle breeds. *Journal of animal breeding and genetics* **2002**, *119*, 361-366.

45. Jiang, Y.; Li, N.; Fan, X.; Xiao, L.; Xiang, R.; Hu, X.; Du, L.; Wu, C. Associations of T→ A mutation in the promoter region of myostatin gene with birth weight in Yorkshire pigs. *Asian-Australasian Journal of Animal Sciences* **2002**, *15*, 1543-1545.
46. Gu, Z.-L.; Zhang, H.-F.; Zhu, D.-H.; Li, H. Single nucleotide polymorphism analysis of the chicken Myostatin gene in different chicken lines. *Yi chuan xue bao= Acta genetica Sinica* **2002**, *29*, 599-606.
47. Kaminski, S. Bovine kappa-casein (CASK) gene-molecular nature and application in dairy cattle breeding. *Journal of Applied Genetics* **1996**, *2*, 176-196.
48. Barroso, A.; Dunner, S.; Cañón, J. Polimorfismo genético de las lactoproteínas de los rumiantes domésticos-Revisión. *ITEA* **1999**, *2*, 143-179.
49. Bernard, C.; Cassar-Malek, I.; Le Cunff, M.; Dubroeucq, H.; Renand, G.; Hocquette, J.-F. New indicators of beef sensory quality revealed by expression of specific genes. *Journal of Agricultural and Food Chemistry* **2007**, *55*, 5229-5237.
50. Glass, D.J. Skeletal muscle hypertrophy and atrophy signaling pathways. *The international journal of biochemistry & cell biology* **2005**, *37*, 1974-1984.
51. Neyama, H.; Hamada, Y.; Tsukahara, R.; Narita, M.; Tsukamoto, K.; Ueda, H. Blockade of analgesic effects following systemic administration of N-methyl-kyotorphin, NMYR and arginine in mice deficient of preproenkephalin or proopiomelanocortin gene. *Peptides* **2018**, *107*, 10-16.
52. Fang, X.; Xu, H.; Zhang, C.; Chen, H.; Hu, X.; Gao, X.; Gu, C.; Yue, W. Polymorphism in BMP4 gene and its association with growth traits in goats. *Molecular biology reports* **2009**, *36*, 1339-1344.
53. Wozney, J.M.; Rosen, V.; Celeste, A.J.; Mitsock, L.M.; Whitters, M.J.; Kriz, R.W.; Hewick, R.M.; Wang, E.A. Novel regulators of bone formation: molecular clones and activities. *Science* **1988**, *242*, 1528-1534.
54. Malan, S. The improved Boer goat. *Small Ruminant Research* **2000**, *36*, 165-170.
55. Lau, P.; Nixon, S.J.; Parton, R.G.; Muscat, G.E. ROR α regulates the expression of genes involved in lipid homeostasis in skeletal muscle cells: Caveolin-3 and CPT-1 are direct targets of ROR. *Journal of Biological Chemistry* **2004**.
56. Silveira, A.C.; Morrison, M.A.; Ji, F.; Xu, H.; Reinecke, J.B.; Adams, S.M.; Arneberg, T.M.; Janssian, M.; Lee, J.-E.; Yuan, Y. Convergence of linkage, gene expression and association data demonstrates the influence of the RAR-related orphan receptor alpha (RORA) gene on neovascular AMD: a systems biology based approach. *Vision research* **2010**, *50*, 698-715.
57. Sevane, N.; Armstrong, E.; Wiener, P.; Wong, R.P.; Dunner, S.; Consortium, G. Polymorphisms in twelve candidate genes are associated with growth, muscle lipid profile and meat quality traits in eleven European cattle breeds. *Molecular biology reports* **2014**, *41*, 4721-4731.
58. Sevane, N.; Crespo, I.; Cañón, J.; Dunner, S. A Primer-Extension Assay for simultaneous use in cattle Genotype Assisted Selection, parentage and traceability analysis. *Livestock Science* **2011**, *137*, 141-150.
59. Thaller, G.; Kühn, C.; Winter, A.; Ewald, G.; Bellmann, O.; Wegner, J.; Zühlke, H.; Fries, R. DGAT1, a new positional and functional candidate gene for intramuscular fat deposition in cattle. *Animal genetics* **2003**, *34*, 354-357.

60. Guan, D.; Luo, N.; Tan, X.; Zhao, Z.; Huang, Y.; Na, R.; Zhang, J.; Zhao, Y. Scanning of selection signature provides a glimpse into important economic traits in goats (Capra hircus). *Scientific Reports* **2016**, *6*, 36372.
61. Fijneman, R.J.; Bade, L.K.; Peham, J.R.; Van De Wiel, M.A.; Van Hinsbergh, V.W.; Meijer, G.A.; O'Sullivan, M.G.; Cormier, R.T. Pla2g2a attenuates colon tumorigenesis in azoxymethane-treated C57BL/6 mice; expression studies reveal Pla2g2a target genes and pathways. *Analytical Cellular Pathology* **2009**, *31*, 345-356.
62. Wu, L.; Wary, K.K.; Revskoy, S.; Gao, X.; Tsang, K.; Komarova, Y.A.; Rehman, J.; Malik, A.B. Histone demethylases KDM4A and KDM4C regulate differentiation of embryonic stem cells to endothelial cells. *Stem cell reports* **2015**, *5*, 10-21.
63. Tong, G.Q.; Heng, B.C.; Tan, L.G.; Ng, S.C. Aberrant profile of gene expression in cloned mouse embryos derived from donor cumulus nuclei. *Cell and tissue research* **2006**, *325*, 231-243.
64. Carlsson, P.; Mahlapuu, M. Forkhead transcription factors: key players in development and metabolism. *Developmental biology* **2002**, *250*, 1-23.
65. Uhlenhaut, N.H.; Treier, M. Foxl2 function in ovarian development. *Molecular genetics and metabolism* **2006**, *88*, 225-234.
66. Vaiman, D.; Koutita, O.; Oustry, A.; Elsen, J.-M.; Manfredi, E.; Fellous, M.; Cribiu, E. Genetic mapping of the autosomal region involved in XX sex-reversal and horn development in goats. *Mammalian Genome* **1996**, *7*, 133-137.
67. Messer, L.A.; Wang, L.; Tuggle, C.K.; Yerle, M.; Chardon, P.; Pomp, D.; Womack, J.E.; Barendse, W.; Crawford, A.M.; Notter, D.R. Mapping of the melatonin receptor la (MTNR1A) gene in pigs, sheep, and cattle. *Mammalian genome* **1997**, *8*, 368-370.
68. Chu, M.; He, Y.; Cheng, D.; Ye, S.; Fang, L.; Wang, J. Association between expression of reproductive seasonality and alleles of melatonin receptor 1A in goats. *Animal reproduction science* **2007**, *101*, 276-284.
69. Migaud, M.; Gavet, S.; Pelletier, J. Partial cloning and polymorphism of the melatonin~ 1~ a (Mel~ 1~ a) receptor gene in two breeds of goat with different reproductive seasonality. *REPRODUCTION-CAMBRIDGE-* **2002**, *124*, 59-64.
70. Shi, L.; Yue, W.; Ren, Y.; Lei, F.; Zhao, J. Sex determination in goat by amplification of the HMG box using duplex PCR. *Animal reproduction science* **2008**, *105*, 398-403.
71. Grzybowski, G.; Prusak, B.; Romaniuk, B. A novel variant of the amelogenin gene (AMEL-X) in cattle and its implications for sex determination. 2006.
72. Saleh, A.A.; Hammoud, M.H.; Dabour, N.A.; Hafez, E.E.; Sharaby, M.A. BMPR-1B, BMP-15 and GDF-9 genes structure and their relationship with litter size in six sheep breeds reared in Egypt. *BMC Research Notes* **2020**, *13*, 215, doi:10.1186/s13104-020-05047-9.
73. Cao, X.; Ling, C.; Liu, Y.; Gu, Y.; Huang, J.; Sun, W. Pleiotropic Gene HMGA2 Regulates Myoblast Proliferation and Affects Body Size of Sheep. *Animals : an open access journal from MDPI* **2024**, *14*, doi:10.3390/ani14182721.
74. Yu, S.; Chu, W.; Zhang, L.; Han, H.; Zhao, R.; Wu, W.; Zhu, J.; Dodson, M.V.; Wei, W.; Liu, H.; et al. Identification of Laying-Related SNP Markers in Geese Using RAD Sequencing. *PLoS One* **2015**, *10*, e0131572, doi:10.1371/journal.pone.0131572.

75. Chang, Y.C.; Manent, J.; Schroeder, J.; Wong, S.F.L.; Hauswirth, G.M.; Shylo, N.A.; Moore, E.L.; Achilleos, A.; Garside, V.; Polo, J.M.; et al. Nr6a1 controls Hox expression dynamics and is a master regulator of vertebrate trunk development. *Nature communications* **2022**, *13*, 7766, doi:10.1038/s41467-022-35303-4.
76. Chang, C.; Lee, S.O.; Wang, R.S.; Yeh, S.; Chang, T.M. Androgen receptor (AR) physiological roles in male and female reproductive systems: lessons learned from AR-knockout mice lacking AR in selective cells. *Biology of reproduction* **2013**, *89*, 21, doi:10.1095/biolreprod.113.109132.
77. Bai, Y.; Li, J.; Zhu, H.; Liu, J.; Dong, S.; Longping, L.; Qu, L.; Chen, H.; Song, X.; Lan, X. Deletion mutation within the goat PPP3CA gene identified by GWAS significantly affects litter size. *Reproduction, Fertility and Development* **2021**, *33*, 476-483, doi:10.1071/RD20337.
78. Meng, Q.; Wang, K.; Liu, X.; Zhou, H.; Xu, L.; Wang, Z.; Fang, M. Identification of growth trait related genes in a Yorkshire purebred pig population by genome wide association studies. *Asian-Australas J Anim Sci* **2017**, *30*, 462-469, doi:10.5713/ajas.16.0548.
79. Carvalho Filho, I.; Arikawa, L.M.; Mota, L.F.M.; Campos, G.S.; Fonseca, L.F.S.; Fernandes Júnior, G.A.; Schenkel, F.S.; Lourenco, D.; Silva, D.A.; Teixeira, C.S.; et al. Genome-wide association study considering genotype-by-environment interaction for productive and reproductive traits using whole-genome sequencing in Nellore cattle. *BMC Genomics* **2024**, *25*, 623, doi:10.1186/s12864-024-10520-x.
80. Zhao, Z.; Chu, Y.; Feng, A.; Zhang, S.; Wu, H.; Li, Z.; Sun, M.; Zhang, L.; Chen, T.; Xu, M. STK3 kinase activation inhibits tumor proliferation through FOXO1-TP53INP1/P21 pathway in esophageal squamous cell carcinoma. *Cellular Oncology* **2024**, *47*, 1295-1314, doi:10.1007/s13402-024-00928-8.
81. Lyu, Z.; Qin, N.; Tyasi, T.; Zhu, H.; Liu, D.; Yuan, S.; Xu, R. The Hippo/MST Pathway Member SAV1 Plays a Suppressive Role in Development of the Prehierarchical Follicles in Hen Ovary. *PLOS ONE* **2016**, *11*, doi:10.1371/journal.pone.0160896.
82. Deng, K.; Li, X.; Liu, Z.; Su, Y.; Sun, X.; Wei, W.; Fan, Y.; Zhang, Y.; Wang, F. IGF2BP2 regulates the proliferation and migration of endometrial stromal cells through the PI3K/AKT/mTOR signaling pathway in Hu sheep. *J Anim Sci* **2024**, *102*, doi:10.1093/jas/skae129.
83. Nguyen, L. Thesis Title: Using transcriptomic and proteomic analysis to characterize the biological basis of puberty in Brahman heifers. *Thesis, University of Queensland* **2018**.
84. Hou, S.-C.; Chan, L.-W.; Chou, Y.-C.; Su, C.-Y.; Chen, X.; Shih, Y.-L.; Tsai, P.-C.; Shen, C.K.J.; Yan, Y.-T. Ankrd17, an ubiquitously expressed ankyrin factor, is essential for the vascular integrity during embryogenesis. *FEBS Letters* **2009**, *583*, 2765-2771, doi:https://doi.org/10.1016/j.febslet.2009.07.025.
85. Saleh, A.A.; Rashad, A.M.A.; Hassanine, N.N.A.M.; Sharaby, M.A. Candidate genes and signature of selection associated with different biological aspects and general characteristics of goat. *Emerging Animal Species* **2022**, *5*, 100013, doi:https://doi.org/10.1016/j.eas.2022.100013.
86. Lim, D.; Kim, N.K.; Lee, S.H.; Park, H.S.; Cho, Y.M.; Chai, H.H.; Kim, H. Characterization of genes for beef marbling based on applying gene coexpression network. *International journal of genomics* **2014**, *2014*, 708562, doi:10.1155/2014/708562.

87. Zhang, Q.; Calus, M.P.; Guldbrandtsen, B.; Lund, M.S.; Sahana, G. Estimation of inbreeding using pedigree, 50k SNP chip genotypes and full sequence data in three cattle breeds. *BMC genetics* **2015**, *16*, 88.
88. Stella, A.; Ajmone-Marsan, P.; Lazzari, B.; Boettcher, P. Identification of selection signatures in cattle breeds selected for dairy production. *Genetics* **2010**, *185*, 1451-1461.
89. Supakorn, C. The important candidate genes in goats—a review. *Walailak J. Sci. Tech* **2009**, *6*, 17-36.
90. Maj, A.; Korczak, M.; Bagnicka, E.; Zwierzchowski, L.; Pierzchała, M. A TG-repeat polymorphism in the 5′-noncoding region of the goat growth hormone receptor gene and search for its association with milk production traits. *Small ruminant research* **2007**, *67*, 279-284.
91. Grosclaude, F.; Mahé, M.-F.; Brignon, G.; Di Stasio, L.; Jeunet, R. A Mendelian polymorphism underlying quantitative variations of goat α s1-casein. *Genetique, selection, evolution* **1987**, *19*, 399.
92. Sacchi, P.; Chessa, S.; Budelli, E.; Bolla, P.; Ceriotti, G.; Soglia, D.; Rasero, R.; Cauvin, E.; Caroli, A. Casein haplotype structure in five Italian goat breeds. *Journal of Dairy Science* **2005**, *88*, 1561-1568.
93. Sztankoova, Z.; Senese, C.; Czernekova, V.; Dudkova, G.; Kott, T.; Matlova, V.; Soldat, J. Genomic analysis of the CSN2 and CSN3 loci in two Czech goat breeds. *Animal Science Papers and Reports* **2005**, *23*, 67-70.
94. Rijnkels, M. Multispecies comparison of the casein gene loci and evolution of casein gene family. *Journal of mammary gland biology and neoplasia* **2002**, *7*, 327-345.
95. Cosenza, G.; Pauciullo, A.; Colimoro, L.; Mancusi, A.; Rando, A.; Di Berardino, D.; Ramunno, L. An SNP in the goat CSN2 promoter region is associated with the absence of β-casein in milk. *Animal genetics* **2007**, *38*, 655-658.
96. Fariello, M.-I.; Servin, B.; Tosser-Klopp, G.; Rupp, R.; Moreno, C.; San Cristobal, M.; Boitard, S.; Consortium, I.S.G. Selection signatures in worldwide sheep populations. *PLoS One* **2014**, *9*, e103813.
97. Lindholm-Perry, A.; Kuehn, L.; Smith, T.; Ferrell, C.; Jenkins, T.; Freetly, H.; Snelling, W. A region on BTA14 that includes the positional candidate genes LYPLA1, XKR4 and TMEM68 is associated with feed intake and growth phenotypes in cattle 1. *Animal genetics* **2012**, *43*, 216-219.
98. Fortes, M.; Lehnert, S.; Bolormaa, S.; Reich, C.; Fordyce, G.; Corbet, N.; Whan, V.; Hawken, R.; Reverter, A. Finding genes for economically important traits: Brahman cattle puberty. *Animal Production Science* **2012**, *52*, 143-150.
99. Martin, P.; Palhière, I.; Maroteau, C.; Bardou, P.; Canale-Tabet, K.; Sarry, J.; Woloszyn, F.; Bertrand-Michel, J.; Racke, I.; Besir, H. A genome scan for milk production traits in dairy goats reveals two new mutations in Dgat1 reducing milk fat content. *Scientific reports* **2017**, *7*, 1872.
100. Salo, A.M.; Cox, H.; Farndon, P.; Moss, C.; Grindulis, H.; Risteli, M.; Robins, S.P.; Myllylä, R. A connective tissue disorder caused by mutations of the lysyl hydroxylase 3 gene. *The American Journal of Human Genetics* **2008**, *83*, 495-503.

101. Sansregret, L.; Nepveu, A. The multiple roles of CUX1: insights from mouse models and cell-based assays. *Gene* **2008**, *412*, 84-94.
102. Rao, C.; Foernzler, D.; Loftus, S.K.; Liu, S.; McPherson, J.D.; Jungers, K.A.; Apte, S.S.; Pavan, W.J.; Beier, D.R. A defect in a novel ADAMTS family member is the cause of the belted white-spotting mutation. *Development* **2003**, *130*, 4665-4672.
103. Hosking, B.M.; Wang, S.M.; Chen, S.L.; Penning, S.; Koopman, P.; Muscat, G.E. SOX18 directly interacts with MEF2C in endothelial cells. *Biochemical and biophysical research communications* **2001**, *287*, 493-500.
104. Badaoui, B.; Manunza, A.; Castelló, A.; D'Andrea, M.; Pilla, F.; Capote, J.; Jordana, J.; Ferrando, A.; Martínez, A.; Cabrera, B. Advantages and limitations of authenticating Palmera goat dairy products by pyrosequencing the melanocortin 1 receptor (MC1R) gene. *Journal of dairy science* **2014**, *97*, 7293-7297.
105. Sulem, P.; Gudbjartsson, D.F.; Stacey, S.N.; Helgason, A.; Rafnar, T.; Magnusson, K.P.; Manolescu, A.; Karason, A.; Palsson, A.; Thorleifsson, G. Genetic determinants of hair, eye and skin pigmentation in Europeans. *Nature genetics* **2007**, *39*, 1443.
106. Han, J.; Kraft, P.; Nan, H.; Guo, Q.; Chen, C.; Qureshi, A.; Hankinson, S.E.; Hu, F.B.; Duffy, D.L.; Zhao, Z.Z. A genome-wide association study identifies novel alleles associated with hair color and skin pigmentation. *PLoS genetics* **2008**, *4*, e1000074.
107. Guo, J.; Tao, H.; Li, P.; Li, L.; Zhong, T.; Wang, L.; Ma, J.; Chen, X.; Song, T.; Zhang, H. Whole-genome sequencing reveals selection signatures associated with important traits in six goat breeds. *Scientific reports* **2018**, *8*, 10405.
108. Sundram, U.; Harvell, J.D.; Rouse, R.V.; Natkunam, Y. Expression of the B-cell proliferation marker MUM1 by melanocytic lesions and comparison with S100, gp100 (HMB45), and MelanA. *Modern pathology* **2003**, *16*, 802.
109. Bertolini, F.; Servin, B.; Talenti, A.; Rochat, E.; Kim, E.S.; Oget, C.; Palhière, I.; Crisà, A.; Catillo, G.; Steri, R. Signatures of selection and environmental adaptation across the goat genome post-domestication. *Genetics Selection Evolution* **2018**, *50*, 57.
110. Hayes, B.J.; Pryce, J.; Chamberlain, A.J.; Bowman, P.J.; Goddard, M.E. Genetic architecture of complex traits and accuracy of genomic prediction: coat colour, milk-fat percentage, and type in Holstein cattle as contrasting model traits. *PLoS Genetics* **2010**, *6*, e1001139.
111. Kim, J.; Hanotte, O.; Mwai, O.A.; Dessie, T.; Bashir, S.; Diallo, B.; Agaba, M.; Kim, K.; Kwak, W.; Sung, S. The genome landscape of indigenous African cattle. *Genome biology* **2017**, *18*, 1-14.
112. Bahbahani, H.; Tijjani, A.; Mukasa, C.; Wragg, D.; Almathen, F.; Nash, O.; Akpa, G.N.; Mbole-Kariuki, M.; Malla, S.; Woolhouse, M. Signatures of selection for environmental adaptation and zebu× taurine hybrid fitness in East African Shorthorn Zebu. *Frontiers in genetics* **2017**, *8*, 68.
113. Bahbahani, H.; Clifford, H.; Wragg, D.; Mbole-Kariuki, M.N.; Van Tassell, C.; Sonstegard, T.; Woolhouse, M.; Hanotte, O. Signatures of positive selection in East African Shorthorn Zebu: A genome-wide single nucleotide polymorphism analysis. *Scientific reports* **2015**, *5*, 11729.

114. Tijjani, A.; Utsunomiya, Y.T.; Ezekwe, A.G.; Nashiru, O.; Hanotte, O. Genome sequence analysis reveals selection signatures in endangered trypanotolerant West African Muturu cattle. *Frontiers in genetics* **2019**, *10*, 442.
115. Liu, D.; Chen, Z.; Zhao, W.; Guo, L.; Sun, H.; Zhu, K.; Liu, G.; Shen, X.; Zhao, X.; Wang, Q. Genome-wide selection signatures detection in Shanghai Holstein cattle population identified genes related to adaption, health and reproduction traits. *BMC genomics* **2021**, *22*, 1-19.
116. Dikmen, S.; Wang, X.z.; Ortega, M.S.; Cole, J.; Null, D.; Hansen, P. Single nucleotide polymorphisms associated with thermoregulation in lactating dairy cows exposed to heat stress. *Journal of Animal Breeding and Genetics* **2015**, *132*, 409-419.
117. Luo, H.; Hu, L.; Brito, L.F.; Dou, J.; Sammad, A.; Chang, Y.; Ma, L.; Guo, G.; Liu, L.; Zhai, L. Weighted single-step GWAS and RNA sequencing reveals key candidate genes associated with physiological indicators of heat stress in Holstein cattle. *Journal of Animal Science and Biotechnology* **2022**, *13*, 108.
118. Luo, H.; Li, X.; Hu, L.; Xu, W.; Chu, Q.; Liu, A.; Guo, G.; Liu, L.; Brito, L.F.; Wang, Y. Genomic analyses and biological validation of candidate genes for rectal temperature as an indicator of heat stress in Holstein cattle. *Journal of Dairy Science* **2021**, *104*, 4441-4451.
119. Edea, Z.; Dadi, H.; Dessie, T.; Uzzaman, M.; Rothschild, M.F.; Kim, E.S.; Sonstegard, T.; Kim, K.S. Genome-wide scan reveals divergent selection among taurine and zebu cattle populations from different regions. *Animal genetics* **2018**, *49*, 550-563.
120. Taye, M.; Lee, W.; Caetano-Anolles, K.; Dessie, T.; Hanotte, O.; Mwai, O.A.; Kemp, S.; Cho, S.; Oh, S.J.; Lee, H.K. Whole genome detection of signature of positive selection in African cattle reveals selection for thermotolerance. *Animal Science Journal* **2017**, *88*, 1889-1901.
121. Saravanan, K.; Panigrahi, M.; Kumar, H.; Parida, S.; Bhushan, B.; Gaur, G.; Dutt, T.; Mishra, B.; Singh, R. Genomic scans for selection signatures revealed candidate genes for adaptation and production traits in a variety of cattle breeds. *Genomics* **2021**, *113*, 955-963.
122. Dikmen, S.; Cole, J.B.; Null, D.J.; Hansen, P.J. Genome-wide association mapping for identification of quantitative trait loci for rectal temperature during heat stress in Holstein cattle. *PloS one* **2013**, *8*, e69202.
123. Zamorano-Algandar, R.; Medrano, J.F.; Thomas, M.G.; Enns, R.M.; Speidel, S.E.; Sánchez-Castro, M.A.; Luna-Nevárez, G.; Leyva-Corona, J.C.; Luna-Nevárez, P. Genetic markers associated with milk production and thermotolerance in Holstein dairy cows managed in a heat-stressed environment. *Biology* **2023**, *12*, 679.
124. Ben-Jemaa, S.; Mastrangelo, S.; Lee, S.-H.; Lee, J.H.; Boussaha, M. Genome-wide scan for selection signatures reveals novel insights into the adaptive capacity in local North African cattle. *Scientific reports* **2020**, *10*, 19466.
125. Sigdel, A.; Abdollahi-Arpanahi, R.; Aguilar, I.; Peñagaricano, F. Whole genome mapping reveals novel genes and pathways involved in milk production under heat stress in US Holstein cows. *Frontiers in genetics* **2019**, *10*, 928.
126. Macciotta, N.; Biffani, S.; Bernabucci, U.; Lacetera, N.; Vitali, A.; Ajmone-Marsan, P.; Nardone, A. Derivation and genome-wide association study of a principal component-based measure of heat tolerance in dairy cattle. *Journal of Dairy Science* **2017**, *100*, 4683-4697.

127. Cheruiyot, E.K.; Haile-Mariam, M.; Cocks, B.G.; MacLeod, I.M.; Xiang, R.; Pryce, J.E. New loci and neuronal pathways for resilience to heat stress in cattle. *Scientific reports* **2021**, *11*, 16619.
128. Hu, L.; Sammad, A.; Zhang, C.; Brito, L.F.; Xu, Q.; Wang, Y. Transcriptome analyses reveal essential roles of alternative splicing regulation in heat-stressed Holstein cows. *International Journal of Molecular Sciences* **2022**, *23*, 10664.
129. Bohlouli, M.; Halli, K.; Yin, T.; Gengler, N.; König, S. Genome-wide associations for heat stress response suggest potential candidate genes underlying milk fatty acid composition in dairy cattle. *Journal of Dairy Science* **2022**, *105*, 3323-3340.
130. Otto, P.I.; Guimarães, S.E.; Verardo, L.L.; Azevedo, A.L.S.; Vandenplas, J.; Sevillano, C.A.; Marques, D.B.; Pires, M.d.F.A.; de Freitas, C.; Verneque, R.S. Genome-wide association studies for heat stress response in Bos taurus× Bos indicus crossbred cattle. *Journal of dairy science* **2019**, *102*, 8148-8158.
131. Worku, D.; Hussen, J.; De Matteis, G.; Schusser, B.; Alhussien, M.N. Candidate genes associated with heat stress and breeding strategies to relieve its effects in dairy cattle: a deeper insight into the genetic architecture and immune response to heat stress. *Frontiers in veterinary science* **2023**, *10*, 1151241, doi:10.3389/fvets.2023.1151241.

Supplementary File S1—Supplementary Tables
